# Pessary for Prevention of Preterm Birth and Perinatal Mortality in Pregnancies with a Short Cervix: Systematic Review and Meta-Analysis of Randomized Controlled Trials

**DOI:** 10.3390/diagnostics15121466

**Published:** 2025-06-09

**Authors:** Henrique Graf Provinciatto, Edward Araujo Júnior, Gustavo Yano Callado, Alan Roberto Hatanaka, Roberto Angelo Fernandes Santos, Evelyn Traina, Gabriela Ubeda Santucci França, Luiza Graça Coutinho, Alan Lebrão de Amorim, Lucas Almeida das Chagas, Rosiane Mattar, Marcelo Santucci França

**Affiliations:** 1 Department of Obstetrics, Paulista School of Medicine-Federal University of São Paulo (EPM-UNIFESP), Sao Paulo 04023-062, Brazil; henriquegpprovinciato@gmail.com (H.G.P.); araujojred@terra.com.br (E.A.J.); alan.hatanaka@unifesp.br (A.R.H.); rafss@hotmail.com (R.A.F.S.); etraina@unifesp.br (E.T.); gabi.ubeda1410@gmail.com (G.U.S.F.); luizagracacoutinho@gmail.com (L.G.C.); alanlebrao@gmail.com (A.L.d.A.); lucaschagas94@hotmail.com (L.A.d.C.); m.franca22@unifesp.br (M.S.F.); 2 Albert Einstein Israelite College of Health Sciences, Albert Einstein Israelite Hospital, Sao Paulo 05653-120, Brazil; gycallado@gmail.com

**Keywords:** cervical pessary, short cervix, perinatal mortality, preterm birth, systematic review

## Abstract

**Objective:** This systematic review and meta-analysis aimed to evaluate the efficacy of cervical pessaries in preventing perinatal mortality and extreme preterm birth in pregnancies characterized by a short cervix. **Methods**: The analysis included data from nine randomized controlled trials (RCTs), incorporating a total of 3813 participants. These studies compared the use of cervical pessaries against standard care or other interventions in preventing preterm births in women with a short cervix, defined as less than 30 mm. The eligibility criteria for the trials included studies on asymptomatic pregnant women with a short cervix. The primary outcomes analyzed were perinatal mortality and the incidence of preterm birth before 28 weeks of gestation. **Results**: The results showed an imprecise effect estimate for perinatal mortality (OR = 0.93; 95% CI: 0.54 to 1.62). Similarly, the risk reduction for preterm birth before 28 weeks was also non-significant (OR = 0.76; 95% CI: 0.49 to 1.15). Substantial heterogeneity was observed among the studies (I^2^ = 62%), suggesting variability in the study results, which could have been influenced by differences in the study design, population, and interventions. **Conclusions**: Although the results were statistically inconclusive and the estimates imprecise, the confidence intervals still span possible benefit and harm. Thus, while the current evidence does not support the routine use of cervical pessaries, it also does not indicate an increased risk of fetal or neonatal mortality.

## 1. Introduction

Preterm birth is a global health issue with a significant variation in incidence across different regions. In 2010, approximately 11% of all live births worldwide were preterm, amounting to 14.9 million infants born before 37 weeks of gestation [1]. Of the 2.8 million neonatal deaths in 2013, 35% were due to complications related to prematurity [2]. The factors contributing to preterm birth are multifaceted, including genetic, environmental, and socioeconomic influences [1]. The current interventions to prevent preterm birth in women with a short cervix include vaginal progesterone, cervical pessary, and cervical cerclage [3,4,5]. Despite numerous interventions aimed at preventing preterm birth, their indications remain limited, particularly for twin pregnancies [6], which are at high risk for prematurity and for which there is no recommended treatment for cervical shortening [7,8]. Therefore, despite advances in neonatal care, preventing preterm birth continues to be a significant challenge.

Cervical insufficiency, characterized by painless cervical dilatation leading to preterm birth without labor or uterine contractions, is one of the most significant contributors to spontaneous preterm births [9]. Transvaginal ultrasound has become essential for identifying vulnerable pregnant women by measuring the cervical length [10,11]. A short cervical length in mid-pregnancy predicts preterm birth by 33% [12,13]. Cervical funneling, indicating the opening of the internal os while the external os remains closed, and intraamniotic fluid sludge (IFS) are additional markers of the risk of spontaneous preterm birth [14]. Berghella et al. [15] demonstrated that cervical funneling detected before 25 weeks had moderate sensitivity and a high predictive negative value for predicting spontaneous preterm delivery. Similarly, Hatanaka et al. [16,17] demonstrated that IFS correlated with a cervical length < 25 mm during the second trimester anomaly scan had an important association with preterm birth before 35 weeks.

Three interventions to prevent preterm birth are in evidence: progesterone supplementation, cervical cerclage, and, more recently, the use of cervical pessaries [3,4,18]. The cervical pessary, a vaginal silicone device, supports the cervix and alters its angle, potentially reducing the risk of preterm birth [19]. Multiple randomized clinical trials (RCTs) have evaluated its efficacy in preventing preterm birth in pregnancies with a short cervix, yielding mixed results [18,20].

In November 2014, researchers started the PROMPT (Prospective Meta-analysis for Pessary Trials), a consistent meta-analysis designed to evaluate the potential benefits of using a pessary for the prevention of preterm birth [21]. While this study holds the promise of providing high-quality evidence regarding the use of pessaries in pregnant women with a short cervix, their efficacy in preventing perinatal mortality and extreme preterm birth remains uncertain. The last systematic review by Wennerholm et al. [22] pointed to a statistical difference in spontaneous preterm birth before 28 weeks, favoring the pessary group, but they did not recommend the device’s use in their conclusion. The P5 trial showed similar results, suggesting that pessaries might protect against perinatal mortality and extreme preterm birth [18]. However, in 2023, Hoffmann et al. [23] published a study indicating an increase in perinatal mortality in the pessary group, an aspect not previously reported. Thus, this study proposes to evaluate all randomized clinical trials focused on pessaries, specifically targeting perinatal mortality and extreme preterm birth, and to develop this systematic review and meta-analysis.

The TOPS trial was the first RCT to demonstrate a significant increase in the risk of preterm birth before 28 weeks and perinatal mortality in singleton pregnancies with a short cervix (<20 mm) [23]. Conversely, the P5 trial demonstrated a significant reduction in extreme preterm birth before 28 weeks and perinatal mortality, presenting over 90% protection at this gestational age and over 60% protection against mortality [18].

As noted before, preterm birth remains a major challenge in Obstetrics, with significant implications for neonatal health. Cervical dysfunction could be a key predictor of preterm birth. The use of cervical pessaries offers a potential preventive measure, but current evidence is mixed. The variability in trial outcomes highlights the need for a more nuanced understanding of which subgroups of women benefit most from this intervention [24].

A meta-analysis can address these gaps by providing a more detailed analysis of the efficacy of pessaries in women with a short cervix and the associated perinatal mortality. Therefore, this meta-analysis aimed to do so, providing a more comprehensive evaluation of the efficacy of pessaries in preventing extreme preterm birth and perinatal mortality in women with a short cervix, with the potential to inform clinical practice and guide future research.

Another objective of the current study was to explore whether the use of pessaries is associated with an increased risk of perinatal mortality. Given the variability of the results across the pessary trials, this meta-analysis aimed to evaluate the efficacy of cervical pessaries compared to the nonuse of pessaries in reducing the risk of perinatal mortality and extreme preterm birth.

The primary objective of this study was to assess the efficacy of cervical pessaries compared to the nonuse of pessaries in pregnancies involving a short cervix (co-treatment permitted). Specifically, the study aimed to determine whether the placement of a cervical pessary during the second trimester is capable of reducing the risk of perinatal mortality (fetal/neonatal death). The secondary objective was to evaluate the impact of cervical pessaries on the risk of extreme preterm birth (<28 weeks’ gestation), which is closely associated with perinatal mortality.

## 2. Methods

### 2.1. Search Strategy

The primary search for eligible trials was conducted on 28 June 2024 through the Medline, Embase, and Cochrane Central databases from inception without filters or language restrictions. The study selection process included an initial screening of titles and abstracts followed by a full-text evaluation and protocol assessment. The searches included identifying published protocols across ClinicalTrials.gov and the International Clinical Trials Registry Platform (ICTRP) using terms such as “pessary” AND “pregnancy”; “pessary” AND “short cervix”; “pessary” AND “cervical length”; and “pessary” AND “preterm.” The searches were updated and reviewed to identify new trials.

### 2.2. Selection Criteria

The studies were selected based on the following eligibility criteria: (1) RCTs (2) comparing a cervical pessary (Arabin, Bioteque, or Ingamed) (3) with the nonuse of a pessary (co-treatment allowed) (4) in asymptomatic participants exhibiting a short cervix (<30 mm) during mid-pregnancy; the study evaluated perinatal mortality (a composite outcome of fetal and neonatal death), any preterm birth less than 28 weeks, or a spontaneous preterm birth at less than 28 weeks of pregnancy. Studies that fit within any of the following categories were excluded: (1) quasi-randomized trials, cluster randomized trials, or cross-over trials; (2) trials in which the pessary had been administered for immediately threatened preterm labor, including the preterm premature rupture of ovular membranes (PPROMs) and/or uterine contractions; trials without a protocol registration; trials registered after their completion; (3) trials that discontinued the use of the pessary before 34 weeks of pregnancy; (4) RCTs comparing pessary versus progesterone, or comparing pessary versus cerclage; (5) retracted articles; (6) overlapping studies; or (7) congress abstracts.

### 2.3. Study Selection and Data Extraction

Two authors (H.G.P. and M.S.F.) independently applied the eligibility criteria and selected the studies for inclusion using the Rayyan software 1.6.0, remaining blind to each other’s decisions. The results were then compared, and any discrepancies were resolved by consulting a third author (E.A.J.).

The data were independently extracted by the author (H.G.P.) using Excel spreadsheets (Microsoft Corp., Redmond, WA, USA) to collect information on the intervention, sample size, number of events for prespecified outcomes, and patient demographic data. The extracted data were then reviewed twice independently by two authors (M.S.F. and A.R.H.) for accuracy. Any discrepancies were resolved through additional review sessions.

### 2.4. Data Management and Statistical Analyses

A random effects model was employed in our pairwise meta-analysis using the restricted estimator maximum likelihood (REML) with the Inverse of Variance. The pooled data were computed through an odds ratio (OR) and 95% confidence intervals (CIs).

The heterogeneity across the trials was assessed using the I^2^ measure, interpreted as follows: <25%, ≥25% to 50%, and >50% indicate low, moderate, and high heterogeneity, respectively. A detailed analysis plan was completed and approved before starting the analysis.

A trial sequential analysis (TSA) was conducted to determine whether the cumulative evidence for preterm birth before 28 weeks and perinatal mortality was sufficiently powered (TSA Module version 9.5.10, Copenhagen Trial Unit, Copenhagen, Denmark). Two-sided testing was performed with a type I error rate of 5%, targeting 80% power and aiming for a 20% risk ratio reduction as the intervention effect. The TSA utilized a random effects model and variance-based heterogeneity correction. Additionally, the diversity-adjusted required information size (RIS) was calculated, representing the number of participants needed in a meta-analysis to detect or refute an intervention effect, and a cumulative sequential Z-score curve was constructed [25].

Subgroup analyses by cervical length and pessary type were planned but not feasible due to the limited stratified data in most of the included studies.

### 2.5. Assessment of Risk of Bias

The quality of randomized controlled trials (RCTs) was assessed using the Cochrane Collaboration’s risk of bias tool for randomized trials (RoB 2 tool). Each study was evaluated on five domains: (1) randomization process; (2) deviations from intended interventions; (3) missing outcome data; (4) measurement of the outcome; and (5) selection of the reported result. The studies were categorized as “Low risk” (if all domains have a low risk of bias), “Some concerns” (if any domain has some concerns), or “High risk” (if any domain has a high risk of bias or if three domains have some concerns). Two independent authors (M.S.F. and A.R.H.) conducted the quality assessment, resolving any disagreements through a consensus.

### 2.6. Protocol Registration

The study protocol was submitted to the International Prospective Register of Systematic Reviews (PROSPERO, CRD42023438811). PRISMA for Systematic Reviews (PRISMA) and the Cochrane Handbook for Systematic Reviews were followed for conducting this meta-analysis [26,27].

## 3. Results

### 3.1. Studies Triage

**Figure 1:**
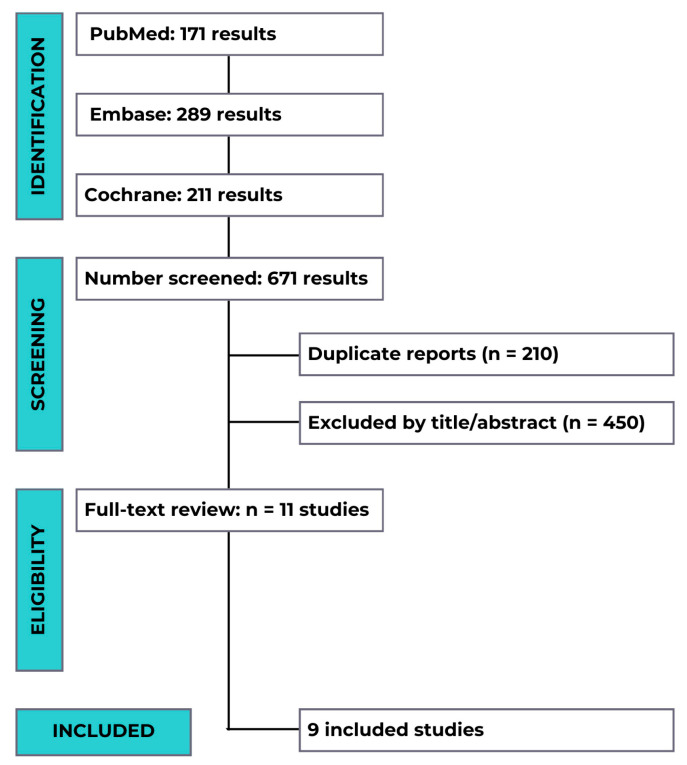
A PRISMA diagram. The flowchart illustrates the systematic review process. It shows the identification from the PubMed, Embase, and Cochrane databases. After the screening and removal of the duplicate reports and the title/abstract, 11 studies were reviewed in full, with 9 studies ultimately included in the analysis.

From our initial search that yielded 671 records, 210 duplicates were removed, leaving 461 records for the title and abstract screening. Of these, 11 studies were fully reviewed based on the predefined eligibility criteria, and their baseline characteristics are exhibited in Figure 1.

Ultimately, the meta-analysis incorporated data from nine randomized controlled trials (RCTs) comparing the use of cervical pessaries versus standard care or other treatments in preventing preterm birth among pregnant women with a short cervix, involving a total of 3813 patients. The total sample size comprised 1936 women in the pessary group and 1877 women in the standard care group.

The studies conducted by Hui et al. [28] (China, ISRCTN11646777) and Karbasian et al. [29] (Iran) were excluded from the meta-analysis because the former had its protocol registered post-trial completion, introducing potential bias, and the latter lacked any protocol registration, undermining the study’s validity and reproducibility.

The baseline characteristics are presented in Table 1 [18,20,23,30,31,32,33,34,35]. Two studies used a 30 mm threshold to define a short cervix, while the remaining studies employed a more stringent cutoff. Among the included studies, three demonstrated significant differences in the baseline cervical length between the intervention and control groups. It is important to note that only randomized controlled trials (RCTs) were included in the study design.

### 3.2. Statistical Results

**Figure 2:**
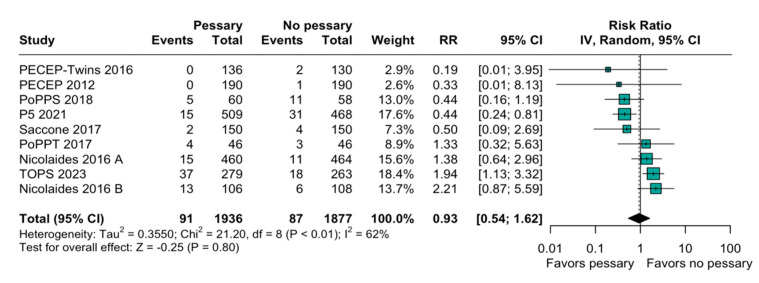
Forest plot of studies included in systematic review with target on perinatal mortality. Forest plot summarizing risk ratio (RR) of perinatal mortality in women using pessary versus no pessary across multiple studies. Overall effect shows inconclusive and imprecise difference (RR = 0.93; 95% CI: 0.54 to 1.62). References in order of appearance in Figure 2 [18,20,23,30,31,32,33,34,35].

**Figure 3:**
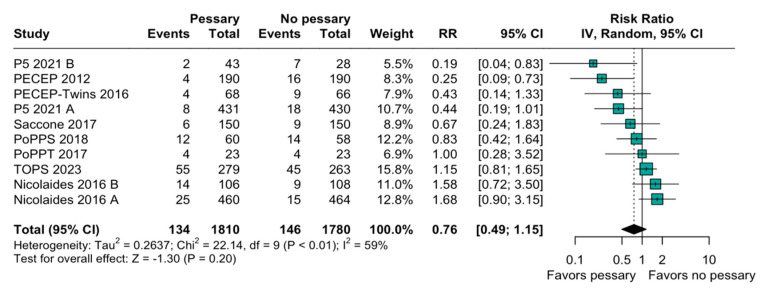
Forest plot of included studies with target on preterm birth below 28 weeks. Forest plot presenting risk ratio (RR) for extreme preterm birth in women using pessary versus no pessary across multiple studies. Overall effect shows inconclusive and imprecise difference (RR = 0.76; 95% CI: 0.49 to 1.15). References in order of appearance in Figure 3 [18,20,23,30,31,32,33,34,35].

**Figure 4:**
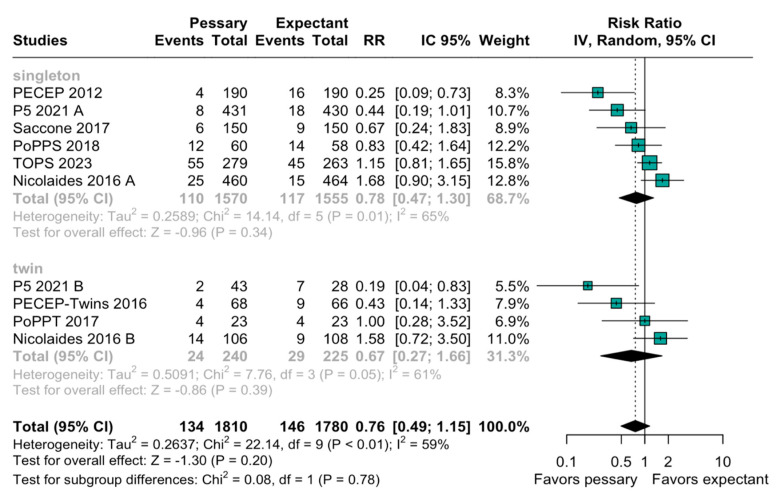
Forest plot of subgroup analysis with singleton and twins with target on preterm birth below 28 weeks. Forest plot presenting risk ratio (RR) for extreme preterm birth in singleton and twin pregnancies using pessary versus expectant management. Overall effect shows inconclusive and imprecise difference in either subgroup. References in order of appearance in Figure 4 [18,20,23,30,31,32,33,34,35].

The relation between the perinatal mortality and pessary was imprecise and does not rule out potential benefits or harm (RR, 0.93; 95% CI: 0.54–1.62; Figure 2). The heterogeneity among the studies was high (I^2^ = 62%), indicating a variability in this outcome. When evaluating preterm birth before 28 weeks, the result was similarly non-significant (RR, 0.76; 95% CI: 0.49–1.15; Figure 3). A subgroup analysis based on the type of pregnancy (singleton or twin) was performed to assess the robustness of the overall findings, and there was an imprecise and statistically inconclusive association, with confidence intervals including both benefit and harm (Figure 4).

**Figure 5:**
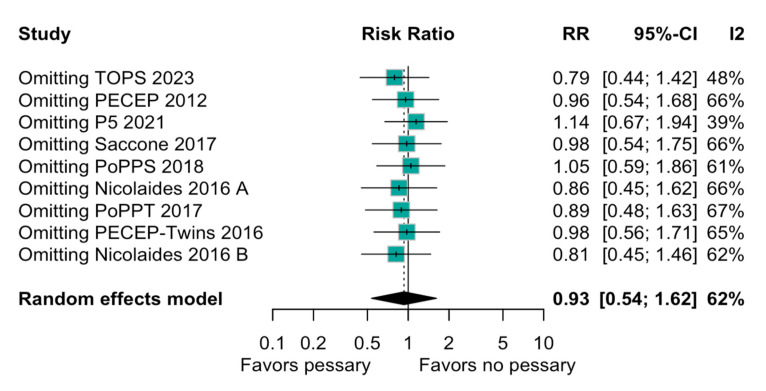
A forest plot of the leave-one-out sensitivity analysis for the perinatal mortality and the use of a pessary in the RCTs included. A forest plot showing the impact of omitting individual studies on the overall risk ratio (RR) for preterm birth with pessary use. The TOPS trial exclusion shows an RR of 0.79 (95% CI: 0.44 to 1.42) for the perinatal mortality when comparing pessary use to the nonuse of a pessary. This suggests a potential reduction in mortality with this exclusion, but the confidence interval is wide, crossing 1, and the heterogeneity (I^2^) suggests moderate variability. References in order of appearance in Figure 5 [18,20,23,30,31,32,33,34,35].

When individual studies were sequentially omitted, the pooled RRs varied slightly but remained consistent with the overall analysis, indicating that no single study disproportionately influenced the results. For example, omitting the TOPS 2023 study resulted in an RR of 0.79 (95% CI: 0.44 to 1.42) with no heterogeneity (I^2^ = 48%), suggesting an increase in the beneficial effect of the pessary in the absence of this study. However, the overall consistency across the analyses supports the robustness of the meta-analysis findings (Figure 5).

**Figure 6:**
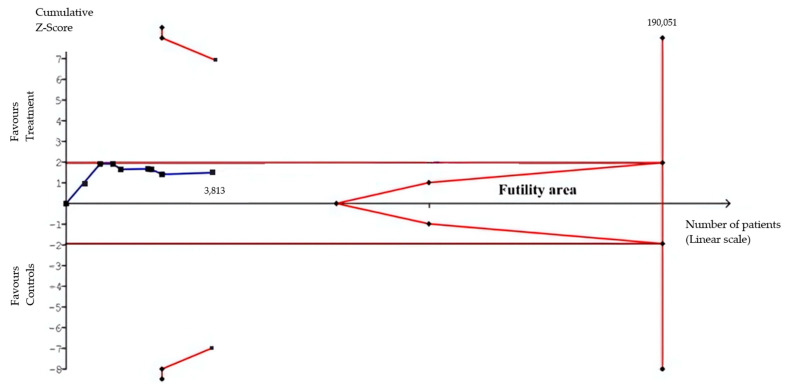
A trial sequential analysis assessing the cumulative evidence for the effect of cervical pessaries on the risk of preterm birth before 28 weeks. The *x*-axis represents the cumulative number of participants, and the *y*-axis shows the Z-statistic over time. The cumulative Z-curve (blue line) did not cross the conventional boundaries (horizontal brown lines) for benefit or the futility boundaries, and the required information size (RIS) was not reached. This indicates that the meta-analysis remains underpowered to draw definitive conclusions, and further studies are needed to clarify the effect.

The TSA for extreme preterm birth did not provide conclusive evidence of a relative risk reduction in the pessary group. The Z-curve did not cross the conventional boundary for benefit or the futility boundary, indicating that the meta-analysis is underpowered for definitive conclusions. Nonetheless, while the cumulative sequential Z-score curve did not cross the conventional boundary for benefit, it also did not surpass the RIS line or the futility boundaries (Figure 6). For perinatal mortality, it was not possible to generate the TSA due to the limited sample size in comparison with the RIS.

### 3.3. Quality Assessment

**Figure 7:**
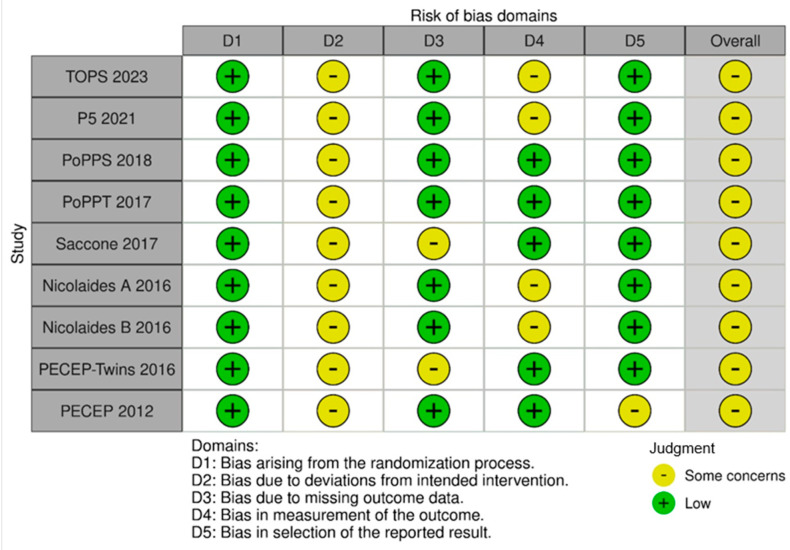
A risk of bias assessment across the five domains for the included studies. This risk of bias table evaluates several studies across five domains: the randomization process (D1), deviations from the intended intervention (D2), missing outcome data (D3), the measurement of the outcome (D4), and the selection of the reported result (D5). The judgments are represented as “Low” (green) or “Some concerns” (yellow). References in order of appearance in Figure 7 [18,20,23,30,31,32,33,34,35].

All of the included RCTs were considered to have some concerns of bias (Figure 7). This analysis considered the open-label characteristic of the intervention, the potential for selective reporting, and differences in the outcomes’ measurement.

## 4. Discussion

This meta-analysis had as its primary objective the assessment of the cervical pessary’s efficacy in diminishing the risk of mortality and extreme preterm birth. The data were obtained from multiple RCTs and are intended to contribute high-quality evidence about the use of pessaries.

### 4.1. Summary of Findings

Varying inclusion criteria and methodologies were combined, resulting in the inclusion of nine studies in this meta-analysis, which indicated that the use of a pessary does not improve efficacy when compared to standard care in the reduction of perinatal mortality. On the other hand, significant variability in the study designs, populations, and interventions was noticed, as considerable heterogeneity was observed across the studies. In addition, a risk ratio (RR) of 0.76 (95% CI: 0.49 to 1.15) (Figure 3) was detected in the secondary analysis, suggesting that the use of a pessary does not significantly impact the rate of extreme preterm birth (<28 weeks). However, in both analyses, it cannot be declared that the use of a pessary increases extreme prematurity or perinatal mortality.

### 4.2. Strengths and Limitations

#### 4.2.1. Strengths

1. Inclusion of only RCTs: Utilizing only randomized controlled trials (RCTs) allows for a more precise and reliable analysis, enabling adjustments for baseline characteristics and the exploration of subgroup effects.
2. Comprehensive Search Strategy: The extensive search strategy across multiple databases and the inclusion of unpublished data ensure a thorough collection of relevant studies.

#### 4.2.2. Limitations

1. Heterogeneity: Prematurity research is inherently heterogeneous due to the global variability in clinical thresholds and practices. Additional factors contributing to the heterogeneity in this meta-analysis include the use of different pessary models, the variability in cervical length thresholds (e.g., <20 mm vs. <30 mm), and the inconsistent reporting of progesterone co-administration. Different pessary types were used across the included trials, which may have influenced the clinical outcomes. However, the absence of stratified outcome data by pessary model limited our ability to assess this effect. Unfortunately, due to the insufficient stratified data in the primary studies, a further subgroup analysis was not feasible.
2. Limited Data on Specific Subgroups: Some subgroups, such as those based on the presence of amniotic sludge, had limited data, making it challenging to draw robust conclusions for these groups.
3. Potential for Bias: Although all of the included studies were RCTs, several exhibited methodological limitations that may affect the reliability of outcome estimates. The open-label design of these trials may have introduced detection bias, particularly for outcomes such as NICU admission or diagnosis timing. This, coupled with concerns regarding the outcome measurements, may have influenced how the outcomes were ascertained and reported. Furthermore, the potential for publication bias and selective reporting cannot be entirely ruled out despite efforts to include unpublished data and perform a comprehensive search strategy.

### 4.3. Evaluation of Pessary Use and Perinatal Mortality

One of the TOPS study’s results was an increase in the perinatal mortality associated with extreme preterm birth with the use of a pessary [23]. However, this meta-analysis does not support this finding. In fact, contrary results can be demonstrated when compared to the cited trial. In the pessary group, both mortality and extreme preterm birth were less evident compared to the standard care, although the results were statistically inconclusive and imprecise.

One hypothesis for the different results between the TOPS trial and this meta-analysis is TOPS’s analytical approach because it combined fetal and neonatal deaths in a single group, evaluating them both as one. When the primary result (preterm birth before 37 weeks) was the only focus, both groups had a 45% rate of preterm birth (OR 1.00 [0.83–1.20]). When considering spontaneous preterm birth only as the main reason to use the cervical pessary, both the pessary and the standard care groups had a 40% preterm birth rate (OR 0.97 [0.79–1.19]). In conclusion, this fact demonstrates that preterm birth was not the principal cause for the observed mortality difference.

In the TOPS trial, the fetal death rate was higher (6.5%) in the pessary group than in the standard care group (3.4%) (OR not presented). Nevertheless, it is important to state that this result was not caused by the pessary, but likely by chance. This effect occurred in the same way in the P5 trial, but in a contrary form. In the P5 trial, the rate of stillbirths was lower in the pessary group (0.8%) than in the standard care group (2.6%) (OR 0.31 [0.10 to 0.97]), and the difference was statistically significant. Nonstatistical adjustments were made to show that progesterone could be the cause of the higher rates of fetal death [18].

The TOPS trial could not conclusively determine that the pessary was the cause of the observed perinatal mortality, as that study was not designed with this specific endpoint in mind. To achieve sufficient statistical power to demonstrate such a finding, the statistical calculations would need to be adjusted to a 99% confidence interval. This adjustment would ensure greater precision and reliability in attributing causality to the pessary, thereby addressing the limitations of the current study design.

In addition, a leave-one-out sensitivity analysis was performed, which excluded in sequence and one-by-one each study from the literature, to recalculate the odds ratio (OR) without the excluded study. This test emphasizes the potential bias introduced by the TOPS trial because when it was excluded, the odds ratio combining the remaining eight studies was calculated as 0.79 (95% CI: 0.44 to 1.42), and the pessary use showed a non-significant increase in its protective effect.

The subgroup analysis for the cervical length suggests a potentially greater benefit for women with more significant cervical shortening, but this finding requires further validation due to the high heterogeneity.

Importantly, it should be noted that the perinatal mortality was not increased with the pessary use, as was demonstrated by the TOPS trial, contrary to previous literature suggesting an indifferent effect with the pessary use.

### 4.4. Interpretation of Findings

The forest plot (Figure 2) comparing the risk ratios (RRs) for perinatal mortality demonstrates a non-significant trend towards a reduction in mortality with the use of a pessary (RR 0.81 [95% CI: 0.59 to 1.11]). The heterogeneity among the studies (I^2^ = 33%) suggests some variability in the studies’ designs, populations, and interventions. The leave-one-out sensitivity analysis (Figure 5) confirms the robustness of the meta-analysis findings by showing that no single study disproportionately influenced the overall results.

These findings are important as they highlight the potential variability and influence of individual studies on meta-analysis outcomes. The non-significant risk ratio reduction and the impossibility of generating a TSA in perinatal mortality could demonstrate that the pessary and non-pessary mortalities were very similar and that both the increased mortality in the progesterone group in the P5 trial and the increased mortality in the pessary group in the TOPS trial were due to chance. The risk ratio analysis and TSA of extreme preterm birth in the pessary group compared to the standard care group suggest a potential benefit that warrants further investigation. The discrepancy in the TOPS trial’s findings compared to other studies underscores the need for careful consideration of study methodologies and analyses when interpreting the results.

While the findings did not reach statistical significance, the overall direction consistently points toward a protective effect of the pessary, particularly in reducing the risk of extreme preterm birth. The lack of precision in the estimates does not dismiss this possibility; instead, it underscores the importance of conducting better-designed trials to clarify the effect. Since the confidence intervals include both potential benefit and harm, clinical decisions should be made on a case-by-case basis. Still, there is no current indication of an increased risk, and the pessary may serve as a useful option in selected clinical settings.

### 4.5. Clinical Implications and Future Research

This meta-analysis suggests that cervical pessaries might help reduce the risk of perinatal mortality and extreme preterm birth, especially when used alongside vaginal progesterone. Although the current evidence is imprecise, the overall direction of the data leans more toward benefit than harm. As such, while its routine use is not yet warranted, considering the pessary in selected cases—guided by individual risk profiles and clinical judgment—appears reasonable.

Future research should focus on conducting high-quality RCTs with homogeneous populations to better understand the efficacy of pessaries in preventing preterm birth and perinatal mortality. Specifically, studies should aim to identify which subgroups of women are most likely to benefit from the pessary use and the optimal timing and duration of the pessary placement. Additionally, further investigation into the potential benefits of combining pessary use with other interventions, such as vaginal progesterone, is warranted.

## 5. Conclusions

This meta-analysis provides valuable insights into the potential role of cervical pessaries in preventing extreme preterm birth and perinatal mortality among women with a short cervix. While the overall effect was statistically inconclusive and imprecise, the trend towards benefit and the findings from the subgroup analyses highlight the need for further research. Although current evidence does not support the routine use of cervical pessaries, it also does not indicate an increased risk of fetal or neonatal mortality.

## Figures and Tables

**Table 1:** Baseline characteristics of included studies.

Study	Inclusion Criteria	Gestational Age at Cervical Screening (Weeks of Pregnancy)	Intervention	Control
PECEP 2012 [34]	Singleton pregnancies with a cervical length of 25 millimeters or less	18 0/7–22 6/7	Arabin pessary + standard care (*n* = 190)	Standard care (no pessary) (*n* = 190)
PECEP-Twins 2016 [35]	Twin pregnancies with a cervical length of 25 millimeters or less	18 0/7–22 6/7	Arabin pessary + standard care (*n* = 68)	Standard care (no pessary) (*n* = 66)
Nicolaides 2016 A [30]	Singleton pregnancies with a cervical length of 25 millimeters or less	20 0/7–24 6/7	Arabin pessary + standard care (*n* = 460)	Standard care (no pessary) (*n* = 464)
Nicolaides 2016 B [20]	Twin pregnancies irrespective of cervical length	20 0/7–24 6/7	Arabin pessary + standard care (*n* = 106)	Standard care (no pessary) (*n* = 108)
Saccone 2017 [31]	Singleton pregnancies with a cervical length of 25 millimeters or less	18 0/7–23 6/7	Arabin pessary + standard care (*n* = 150)	Standard care (no pessary) (*n* = 150)
PoPPT 2017 [32]	Twin pregnancies with a cervical length of 30 millimeters or less	18 0/7–23 6/7	Bioteque pessary + standard care (*n* = 23)	Standard care (no pessary) (*n* = 23)
PoPPS 2018 [33]	Singleton pregnancies with a cervical length of 25 millimeters or less	18 0/7–23 6/7	Bioteque pessary + standard care (*n* = 60)	Standard care (no pessary) (*n* = 58)
TOPS 2023 [23]	Singleton pregnancies with a cervical length of 20 millimeters or less	16 0/7–23 6/7	Arabin pessary + standard care (*n* = 279)	Standard care (no pessary) (*n* = 263)
P5 2021 [18]	Singleton or twin pregnancies with a cervical length of 30 millimeters or less	18 0/7–22 6/7	Ingamed pessary + vaginal progesterone (200 mg/d) (*n* = 431)	Vaginal progesterone (200 mg/d) (*n* = 430)

## Data Availability

The data presented in this study are available on request from the corresponding author.
